# Mature teratoma treated as lymphatic malformation for 5 years: a case report and literature review

**DOI:** 10.1080/23320885.2019.1619458

**Published:** 2019-05-30

**Authors:** Hayato Maruguchi, Tadashi Nomura, Satoru Sasaki, Kazunobu Hashikawa, Hiroto Terashi

**Affiliations:** a Department of Plastic Surgery, Kobe University Graduate School of Medicine, Kobe, Hyogo, Japan;; b Center for Vascular Anomalies, Tonan Hospital, Sapporo, Hokkaido, Japan

**Keywords:** Teratoma, lymphatic malformation, congenital neck mass, sclerotherapy, OK-432

## Abstract

Teratomas and lymphatic malformations are included in the differential diagnosis of congenital neck masses. They can exhibit similar clinical findings. The authors present a case of mature teratoma that had been managed as a lymphatic malformation for years. Clinicians should be careful not to dismiss clues for the correct diagnosis.

## Introduction

Teratomas are germ-cell tumors composed of tissues derived from multiple germ-cell layers. They occasionally develop in cervical lesions, and are included in the differential diagnosis of congenital neck masses. Lymphatic malformations (LMs) are also one of these differentials; however, their physical and imaging findings are occasionally similar and can be confused with one another.

OK-432 sclerotherapy is one treatment used to shrink the cystic component of LMs. Because of their similarity, misdiagnosed teratomas may undergo sclerotherapy, which is assumed to be ineffective. We present a rare case of pharynx and parotid gland mature teratoma, which had been shrunk by multiple sclerotherapy sessions until the patient was five years of age. Due to an uncommon medical history and reaction to sclerotherapy, this teratoma was believed to be an LM until surgical resection demonstrated otherwise when the patient was six years of age. We reviewed the literature to determine the appropriate approach to clinical diagnosis.

## Case presentation

**Figure 1.**
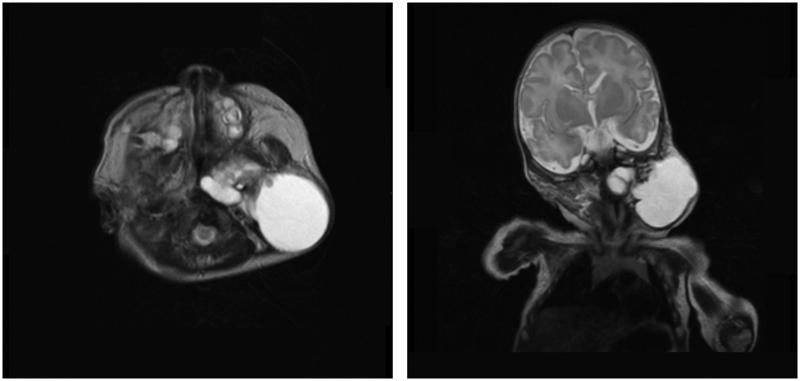
T2 weighted MRI at 1-month-old. The mass was multicystic and located near pharynx and parotid gland.

**Figure 2.**
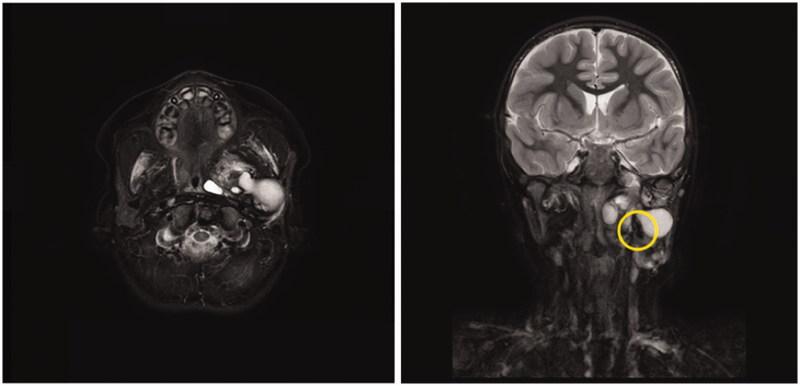
Short-tau inversion recovery (STIR) MRI at 5 years old. Majority of cystic components had shrinked. Hypo-intense structure beside the cyst (circle) was figured out to be calcification during operation later.

An infant boy with a congenital left neck mass was referred to pediatricians at a local university hospital. Magnetic resonance imaging (MRI) revealed a multi-cystic mass located near the pharynx and parotid gland (Figure 1). This patient’s history, physical examination, and MRI findings suggested LM. He underwent OK-432 sclerotherapy at 2, 3 and 6 months of age, which successfully reduced the volume of the mass as expected each time. He grew without respiratory or feeding issues, except snoring while he slept. Because the bulge on his left neck persisted, a fourth sclerotherapy procedure was performed for cosmetic reasons when the patient was 3 years of age. The doctors could not reach the cystic component using aspiration and, thus, relinquished sclerotherapy and consulted an experienced plastic surgeon for further treatment. MRI performed at that time revealed that the majority of the mass consisted of thick, wall-like tissue with a small cystic component (Figure 2). The plastic surgeon attempted sclerotherapy once again; however, the cyst wall was so thick that he concluded further shrinkage using sclerotherapy would be futile.

The surgeon recommended surgical resection as an LM, and the patient was referred to the authors’ department at 5 years of age. Ultrasound (US) revealed virtually no cystic component and no fluid flow, which is consistent with LM, scar tissue, and blood clots due to previous sclerotherapy.

**Figure 3.**
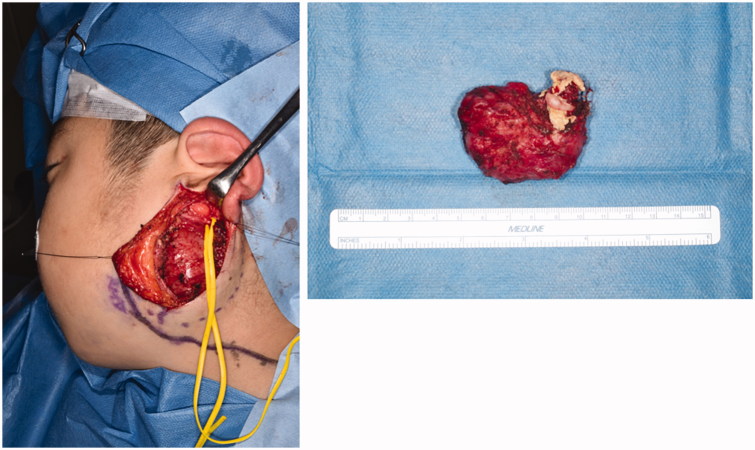
Intraoperative findings. The mass was smooth and located between parotid gland and sternocleidomastoid muscle. It was composed of cystic and solid components with bone- and tooth-like tissue, hair, and sebaceous sebum matter.

When the patient was 6 years of age, surgical resection was planned. Intra-operatively, a smooth cystic mass was found between the parotid gland and sternocleidomastoid muscle. The mass was aspirated directly to deflate it with the aim of easier resection. The internal liquid was dull beige in color. The base of the mass was in contact with the internal carotid artery and vein. The mass was completely excised. On examination, it was composed of cystic and solid components, with bone- and tooth-like tissue, hair, and sebaceous sebum matter (Figure 3).

**Figure 4.**
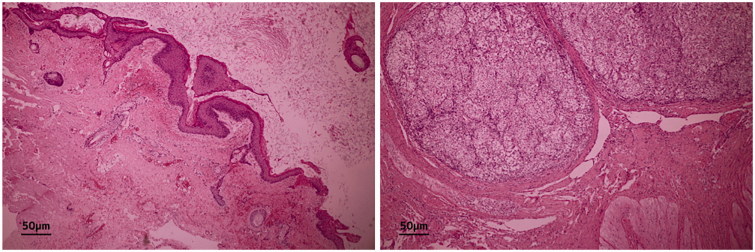
Pathological findings. Pathological exam finds skin with accessory glands and adipose tissue, choroid tissue, anterior pituitary gland tissue. The pathological diagnosis is mature teratoma.

Cytological examination of the internal liquid revealed squamous and inflammatory cells and necrotic material. On pathological examination, the mass contained choroid and anterior pituitary gland tissue, skin with accessory glands, and adipose tissue (Figure 4). No immature tissue components were observed. Based on these findings, the mass was diagnosed as a mature teratoma. The patient is under observation on an outpatient basis.

## Discussion

Differential diagnosis of congenital neck masses include variety of diseases. They are classified in three categories: inflammatory, neoplastic and congenital. Congenital neck masses include branchial cleft cysts, thyroglossal duct cysts, laryngocele, ranula, dermoid cyst, and thymic cyst [1]; teratomas and LMs are also included.

Teratomas are a type of germ-cell tumor, and more than one-half are located in the sacrococcygeal area, followed by the ovary, testis, mediastinum, retroperitoneum, and cervical lesions. Cervical teratomas are uncommon and account for less than 3-5% [2,3] of all teratomas, and only 2% of all congenital neck masses [1]. They are often related to the thyroid and located at the midline area [4]. Teratomas are generally benign, but they are known to undergo malignant transformation when left untreated; thus, surgical removal is recommended [5–7].

LMs are benign, slow-flowing vascular lesions composed of dilated lymphatic channels or cysts lined with endothelial cells with a lymphatic phenotype. They are classified as macrocystic, microcystic, or mixed type according to their structure, and often develop in the cervicofacial and axillary area [8]. OK-432 sclerotherapy is generally preferred for macrocystic LMs to address cosmetic and functional issues. Surgical resection can be a therapeutic option if appropriate.

The relatively low incidence of teratomas and high incidence of LMs, and their similar clinical characteristics make it difficult to distinguish teratomas from LMs [9]. The diagnosis is more confusing when the mass is located in a lateral cervical lesion where LMs are common and teratomas are rare. Clinical characteristics of cervical teratomas and LMs are summarized in Table 1.

**Table 1.** Clinical characteristics of cervical teratomas and LMs.

	Cervical teratomas	Cervical LMs
Percentage in congenital neck mass	2%	6%
Malignancy	Possible	Never
Most frequent area of neck	Midline area	Lateral area
Structural characteristics	Cystic and/or solid calcification(not always)	Multicystic and/or macrocystic
Method of definitive diagnosis	Pathological examination	Pathological examination
Common therapy	Surgical resection	Sclerotherapy / surgical resection

As noted above, surgical removal should be first-line therapy if teratomas is suspicious. However, previous studies present clinical course of misdiagnosed cystic teratomas, which have been treated by OK-432 sclerotherapy as LMs. [4,10–13] (Table 2). The majority of these reports claim that sclerotherapy did not shrink the cystic components. Unexpected and disappointing result prompted surgical resection or tissue biopsy to make a correct diagnosis in these cases.

**Table 2.** List of teratoma patients underwent OK-432 sclerotherapy as lymphatic malformation.

Patient	Gender	Midline/lateral	Site	Initial image findings	Age at correct diagnosis	Method of diagnosis	Maturity	Number of sclerotherapy	Shrinkage by sclerotherapy
#1	Male	Lateral	Pharynx and parotid gland	Multilocular cyst	6 years	Complete resection	Mature	5	YES
#2 [4]	Male	Lateral	Neck ∼ buccal area	Unilocular cyst	8 months	Complete resection	Partially immature	3	NO
#3 [10]	Female	Lateral	Neck ∼ skull base	Unilocular cyst	6 months	Partial resection	Unknown	2	NO
#4 [11]	Unknown	Midline ∼ lateral	Neck ∼ chest	Multilocular cyst	Several months	Complete resection	Mature	2	NO
#5 [12]	Male	Lateral	Neck ∼ mandibular	Multilocular cyst	2 years 6 months	Complete resection	Mature	17	NO
#6 [15]	Female	Lateral	Parapharyngeal space ∼ skull base	Unilocular cyst	2 years 11 months	Biopsy	Mature (+yolk sac tumor)	8	YES

Sclerotherapy is not a radical treatment for teratoma. Moreover, it can make surgical resection difficult due to the scar tissue left behind. Because early removal is advised for teratoma, it is preferred to make a definitive diagnosis to clearly distinguish teratoma from LM before intervention. We reviewed the literature to determine the characteristics of provisional examinations.

## Diagnostic imaging

US is a convenient and popular method for initial assessment, while CT and MRI are both useful to evaluate the mass, in whole or in part, in different ways.

Teratomas can be cystic, solid, or a combination of both, with proportions of each being variable. A mixed cystic/solid lesion highly suggests teratoma [2]. On US, heterogeneous masses with solid and cystic components are highlighted. On CT, hypo-attenuation and heterogeneous masses, with or without calcification, are usually observed. On MRI, the cystic components of a teratoma are often hypo- to iso-intense on T1-weighted imaging, and hyper-intense on T2-weighted imaging. Fat suppressed T2-weighted imaging and short-tau inversion recovery imaging are preferred to characterize the extent of the mass [14]. However, all of these imaging findings are variable, reflecting the wide variety of internal components. Teratomas may contain characteristic tissue-like hair or teeth, which increase the likelihood of presence if detected.

LMs usually present as a cystic lesion filled with lymphatic fluid. They are classified as macrocystic or microcystic according to structure. US reveals cystic lesions with a cyst wall of variable thickness. On CT, they appear as hypo-attenuated polycystic masses. On MRI, cystic components are hypo- to iso-intense on T1-weighted imaging, and hyper-intense on T2-weighted imaging. MRI imaging is the most reliable modality for characterizing the extent of the mass [14]. However, these finding are altered by internal hemorrhage and infection.

In the case presented, multiple sclerotherapy sessions had modified the structure of the teratoma. Even though mixed cystic/solid lesion suggests teratoma [2], the solid component of this teratoma was disguised as remaining solid tissue of an LM. Incorrect therapeutic interventions disturbed appropriate assessment and correct diagnosis. However, a closer look at MRI images based on intraoperative findings can highlight calcifications, which should have let us proceed to CT scan, cytology and surgical removal (Figure 2).

In summary, there can be virtually no differences between LMs and teratoma, especially when majority of the mass is composed of cystic lesion and no calcification. Small specific findings may exist; thus, careful examination is essential. We believe MRI is an appropriate option as initial clinical imaging for congenital neck mass, in order to avoid radiation exposure. However, in this case we presented, we should have taken a closer look at MRI and should have noticed small low intensity area that suggests calcification which indicates teratoma.

## Cytology

Cytology is an assistive tool for diagnosis. Cells derived from multiple germ-cell layers usually indicate teratoma. However, puncture fluid consistent with lymph fluid is non-specific. A previous report described a case of teratoma in which repeated puncture fluid cytologies were consistent with lymph, and appropriate intervention was delayed until the patient was 3 years of age [15].

To our knowledge, the specificity and sensitivity of cytology for teratoma and LM remain unclear. However, it makes sense that cells from more than 2 germ-cell layers are highly sensitive for teratoma. Because clinicians cannot diagnose LMs definitively without biopsy or surgical resection, it makes sense to conduct cytology and sclerotherapy at the same time and to confirm the diagnosis of LMs or to obtain clues for correct diagnosis. Cytology can be repeated to increase sensitivity for teratoma; however, its cost effectiveness is unknown. In our case, cytological results of the internal liquid, which contained squamous cells, should have raised the possibility of teratoma if the internal liquid was obtained by skin puncture.

## Reaction to sclerotherapy

As noted above, teratomas tend to be misdiagnosed as other congenital neck masses, including LMs. To our knowledge, OK-432 sclerotherapy was performed for five cases of neck teratoma in the past due to initial misdiagnosis. In four of these cases, OK-432 did not affect the volume of the mass [4,10–12]. However, in one case, it shrunk the cystic lesion 5 times, followed by ineffective results in 3 sessions [15]. This clinical course is similar to the patient described in the present report, whose teratoma had not been diagnosed until 2 years and 11 months of age, when the predominantly solid mass had grown rapidly.

OK-432 sclerotherapy provokes a strong local immunological reaction by many kinds of cytokines [16], which leads to regional adhesion. It is used to shrink cystic lesions, including LMs, taking advantage of this effect.

Ohtas et al attempted OK-432 sclerotherapy in 148 cases of otolaryngological cystic disease (ranulas, lymphatic malformation, branchial cleft cysts, thyroglossal duct cysts, thyroid cysts, and cervical lymphocele), and reported that it was effective in 76% of all cases [17]. The authors also reported it was significantly less effective for branchial cleft cysts, which consists of stratified squamous epithelium. They inferred that the different internal epithelium results in different reactions to therapy. According to this hypothesis, OK-432 may be less effective in teratomas with an internal epithelium with stratified squamous epithelium.

However, past systematic review shows only 83% of LMs decrease in size after sclerotherapy [18]. To put it the other way, 17% of LMs do not shrink after sclerotherapy. Even though teratomas are expected to be less effective to sclerotherapy, clinicians cannot rule out or rule in LMs or teratoma only based on the result of OK-432 sclerotherapy.

## Conclusion

Congenital neck teratomas can be similar to lymphatic malformations in clinical history, physical examination and diagnostic imaging findings, which lack high-specificity or high-sensitivity clues in most cases. Occasionally, they are ‘treated’ by OK-432 sclerotherapy, and even shrink as a result, which makes diagnosis even more difficult.

Pathological evaluation is essential for definitive diagnosis of teratoma or most of the congenital neck mass; thus, careful attention is required in clinical history, examination, radiological findings. Biopsy or resection should be considered when diagnosis is suspicious.
